# Apoptosis of stem cells likely determines the manifestation of type 2 diabetic nephropathy: predictive and preventive potentials

**DOI:** 10.1186/1878-5085-5-S1-A67

**Published:** 2014-02-11

**Authors:** Mahmood S Mozaffari, Jun Yao Liu, Jack C Yu, Babak Baban

**Affiliations:** 1 Department of Oral Biology and Plastic Surgery, Georgia Regents University, Augusta, Georgia 30912, USA

**Keywords:** diabetic nephropathy, stems cells, blood, kidney, predictive and preventive strategies

## Scientific objective

One of the most pressing health-related challenges is the worldwide epidemic of type 2 diabetes and associated complications such as nephropathy. Indeed, type 2 diabetic patients account for the majority of individuals with chronic kidney disease requiring renal replacement therapy. This not only suggests inadequacy of current drug therapies but also failure of endogenous protective mechanisms. Stem cells are known to play pivotal roles in repair and regeneration of damaged/dysfunctional tissues. Our initial observations suggested that initiation and progression of type 2 diabetes mellitus is associated with progressive reduction in endothelial progenitor cells in the obese diabetic db/db mice. Thus, the present study was intended to more fully explore the status of components of stem cells in the setting of type 2 diabetic nephropathy and whether the diabetic milieu affects the survivability of these cells.

## Technical approach/methods

Male 16-week-old obese diabetic db/db mice (and their db/m controls) were used for this study. Accordingly, indices of glycemic status (e.g., insulin resistance index, hemoglobin A1c), kidney function (e.g., albuminuria, creatinine clearance) were determined in the context of assessment of components of stem cells in peripheral blood and renal tissue. In addition, we measured apoptotic cell death in components of stem cells.

## Results/interpretation

The db/db mice showed significant increases in insulin resistance index and hemoglobin A1c in association with marked increase in albuminuria but reduction in creatinine clearance in association with significant increases in apoptotic and necrotic cell death of kidney cells. The peripheral blood cells of db/db mice displayed significant reduction in hematopoietic stem cells (HSC; Sca1^+^Ckit^+^CD31^-^) mesenchymal stem cells (MSCs; Sca1^+^, CD105^+^, CD31^-^) and endothelial/epithelial progenitor cells (EPCs; Sca1^+^Ckit^+^CD31^+^) compared to those of db/m controls. Further, kidney cells prepared for experimental groups also showed reductions in components of stem cells. Using annexin V in association with markers of each component of stem cells revealed significant increases in apoptosis for HSC, MSC and EPCs in cells prepared for kidneys of db/db and db/m mice. Collectively, the results suggest that apoptosis of components of stem cells likely contributes to eventual manifestation of renal failure in type 2 diabetes mellitus.

## Outlook/expert recommendations

The observation of a similar pattern of decline in stem cell components in the peripheral blood and the kidney suggests that monitoring of blood levels of stem cells subsets may be predictive of failure of their renoprotective features. Further, strategies aimed at reducing apoptosis of stem cell components and “empowering” them through genetic modulations could potentially serve as effective strategies to prevent end-stage renal failure in type 2 diabetic patients.

